# Systematic pan-cancer analysis identifies *SLC31A1* as a biomarker in multiple tumor types

**DOI:** 10.1186/s12920-023-01489-9

**Published:** 2023-03-27

**Authors:** Fan-Sheng Kong, Chun-Yan Ren, Ruofan Jia, Yuan Zhou, Jian-Huan Chen, Yaping Ma

**Affiliations:** 1 Department of Pediatrics, Affiliated Hospital of Jiangnan University, Wuxi, 214122 Jiangsu China; 2 Laboratory of Genomic and Precision Medicine, Wuxi School of Medicine, Jiangnan University, Wuxi, Jiangsu China; 3 Joint Primate Research Center for Chronic Diseases, Institute of Zoology of Guangdong Academy of Science, Jiangnan University, Wuxi, Jiangsu China; 4 Jiangnan University Brain Institute, Wuxi, Jiangsu China

**Keywords:** *SLC31A1*, Cuproptosis, Pan-cancer analysis, Biomarker, Immune infiltration, Prognosis

## Abstract

**Background:**

Solute Carrier Family 31 Member 1 (*SLC31A1*) has recently been identified as a cuproptosis-regulatory gene. Recent studies have indicated that *SLC31A1* may play a role in colorectal and lung cancer tumorigenesis. However, the role of *SLC31A1* and its cuproptosis-regulatory functions in multiple tumor types remains to be further elucidated.

**Methods:**

Online websites and datasets such as HPA, TIMER2, GEPIA, OncoVar, and cProSite were used to extract data on *SLC31A1* in multiple cancers. DAVID and BioGRID were used to conduct functional analysis and construct the protein–protein interaction (PPI) network, respectively. The protein expression data of SLC31A1 was obtained from the cProSite database.

**Results:**

The Cancer Genome Atlas (TCGA) datasets showed increased *SLC31A1* expression in tumor tissues compared with non-tumor tissues in most tumor types. In patients with tumor types including adrenocortical carcinoma, low-grade glioma, or mesothelioma, higher *SLC31A1* expression was associated with shorter overall survival and disease-free survival. S105Y was the most prevalent point mutation in *SLC31A1* in TCGA pan-cancer datasets. Moreover, *SLC31A1* expression was positively correlated with the infiltration of immune cells such as macrophages and neutrophils in tumor tissues in several tumor types. Functional enrichment analysis showed that *SLC31A1* co-expressed genes were involved in protein binding, integral components of the membrane, metabolic pathways, protein processing, and endoplasmic reticulum. Copper Chaperone For Superoxide Dismutase, Phosphatidylinositol-4,5-Bisphosphate 3-Kinase Catalytic Subunit Alpha and Solute Carrier Family 31 Member 2 were copper homeostasis-regulated genes shown in the PPI network, and their expression was positively correlated with *SLC31A1*. Analysis showed there was a correlation between SLC31A1 protein and mRNA in various tumors.

**Conclusions:**

These findings demonstrated that *SLC31A1* is associated with multiple tumor types and disease prognosis. *SLC31A1* may be a potential key biomarker and therapeutic target in cancers.

**Supplementary Information:**

The online version contains supplementary material available at 10.1186/s12920-023-01489-9.

## Introduction

Cancer is the leading cause of mortality worldwide, imposing substantial healthcare and socio-economic burden [1]. The treatment strategies for cancer mainly include surgery, chemotherapy, radiotherapy, targeted therapy, and immunotherapy [2–5]. Despite drug resistance, side effects, and other unelucidated issues, the prognosis and survival rate remain unsatisfactory [6]. Recently large-scale and multi-omic pan-cancer studies and databases, such as the Cancer Genome Atlas (TCGA), have made it possible to investigate both the common features and heterogeneities across various human tumors [7–12].

Solute Carrier Family 31 Member 1 (*SLC31A1*), also known as copper (Cu) transporter 1 (CTR1), is considered a key component in cellular Cu uptake in mammalian cells and tissues [13]. Moreover, *SLC31A1* was recently identified as a cuproptosis-regulatory gene, and a high *SLC31A1* expression level can cause Cu-induced cell death [14]. In addition, *SLC31A1* transports platinum drugs across the plasma membrane, and in patients with non-small cell lung cancer, *SLC31A1* is a potential pharmacogenetic biomarker for clinical outcomes [15, 16]. To date, however, there is no comprehensive pan-cancer study of the function and clinical significance of *SLC31A1*.

In our current study, we systematically described the mRNA and protein expression levels, prognostic value, genetic alterations, molecular function of *SLC31A1* in several tumor types as well as the association with immune infiltration. Our findings reveal that *SLC31A1* could be a potential biomarker and novel therapeutic target  of multiple tumors.

## Materials and methods

### Expression analysis

Expression data of *SLC31A1* mRNA was obtained from the Human Protein Atlas (HPA) database (version: 21.1) (https://www.proteinatlas.org) [17]. In multiple tumor types, the “Gene DE” module of Tumor Immune Estimation Resource version 2 (TIMER2) (http://timer.cistrome.org/) was used to investigate *SLC31A1* expression levels in tumors and non-tumor tissues [18–20]. The protein expression level of SLC31A1 was obtained from HPA.

## Prognostic analysis

Kaplan–Meier (K–M) survival analysis of *SLC31A1* for overall survival (OS) and disease-free survival (DFS) was conducted using the Gene Expression Profiling Interactive Analysis version 2 (GEPIA2) (http://gepia2.cancer-pku.cn/) database [21].

## Genetic mutations analysis

We analyzed the characteristics of *SLC31A1* genetic alterations in cBioPortal (v4.1.9) (https://www.cbioportal.org/) [22, 23]. In the "Cancer Types Summary" module, we calculated the frequency of *SLC31A1* gene alterations based on TCGA Pan-Cancer Atlas Studies datasets. The "Mutations" module was used to generate a mutation site plot of *SLC31A1*. Then to confirm the driver mutations in *SLC31A1*, the platform OncoVar (https://oncovar.org/) was used [24]. And the database the Catalogue of Somatic Mutations in Cancer (COSMIC) (https://cancer.sanger.ac.uk) was used to annotate *SLC31A1* somatic mutations [25]. Driver mutations were defined as somatic missense mutations with AI-Driver score ≥ 0.95 and occurred in at least two patients. The International Cancer Genome Consortium (ICGC) (https://dcc.icgc.org/) database was used to confirm the mutation site of SLC31A1 and explore the cancer distribution of *SLC31A1* [26].

## Immune infiltration evaluation

The "Immune" module of TIMER2 was used to analyze the correlation between *SLC31A1* expression and 21 immune infiltrations, including B cells, cancer associate fibroblast, common lymphoid progenitor, common myeloid progenitor, DC, endothelial cells, eosinophil, granulocyte-monocyte progenitor, hematopoietic stem cells, macrophage, mast cells, monocyte, myeloid-derived suppressor cells, neutrophil, NK cells, CD4 + T cells, CD8 + T cells, T cell follicular helper, T cell gamma delta, NK T cells, and Tregs, Several immune deconvolution algorithms were applied, including TIMER, xCell, MCP-counter, CIBERSORT, EPIC, quanTIseq, and CIBERSORT-ABS. Consistent significant findings (*P* < 0.05) by all available algorithms were required to support an accurate correlation with immune infiltrations.

## Gene enrichment analysis and protein interaction network construction

A list of the top 100 genes correlated with *SLC31A1* that had similar expression patterns ranked by Pearson correlation coefficient was obtained from TCGA datasets using the GEPIA2 "Similar Gene Detection" module. In the meantime, Gene Ontology pathway enrichment analysis and Kyoto Encyclopedia of Genes and Genomes (KEGG) analysis were retrieved from the Database for Annotation, Visualization, and Integrated Discovery (http://david.abcc.ncifcrf.gov/) [27–30]. With multiple test correlations, FDR < 0.05 were set as the significance threshold. In addition, pairwise gene correlation analysis was performed using the GEPIA2 “Correlation Analysis” module for all tumor tissues in TCGA. *SLC31A1*-interactive protein networks were constructed with the "Network" module of BioGRID (version. 4.4.216) (https://thebiogrid.org/) [31].

## Relative protein abundance analysis of SLC31A1

The expression data of the relative protein abundance of SLC31A1 was downloaded from Cancer Proteogenomic Data Analysis Site (cProSite) database (https://cprosite.ccr.cancer.gov/). And the correlation of SLC31A1 between relative abundance and mRNA was calculated using the cProSite website.

## Statistical analysis

The statistical analysis was automatically computed based on the above online databases. Student's *t*-test implemented by GraphPad Prism (Version 9.1.1) was used to compare protein expression between tumor tissues and adjacent normal tissues.

## Results

### *SLC31A1* expression in various tissues and tumors

**Fig. 1:**
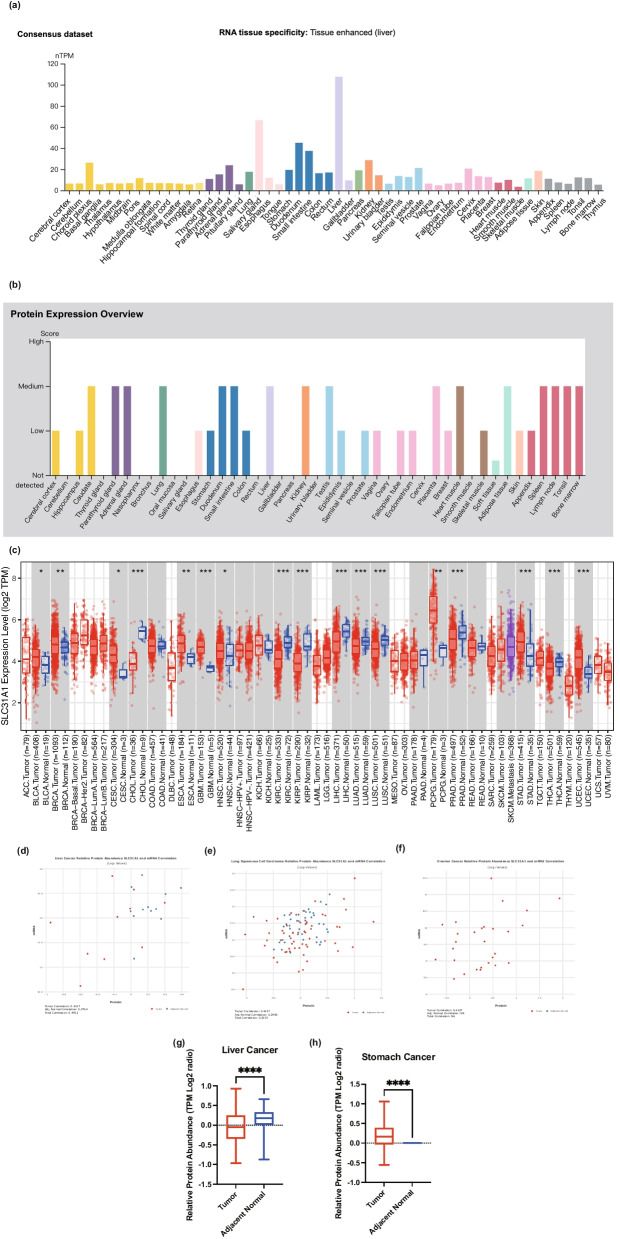
The expression of *SLC31A1* in normal tissues and different tumors. **a** Consensus *SLC31A1* tissue expression based on the consensus dataset in HPA. The X-axis shows tissue or organ types. The Y-axis shows the *SLC31A1* mRNA expression level. nTPM, normalized transcripts per million. **b** The protein expression stare of SLC31A1 in different tissues. The X-axis shows tissues or organ types. The Y-axis shows the SLC31A1 protein expression level. **c** The expression status of *SLC31A1* in different tumor types is visualized by TIMER2. The Y-axis shows the *SLC31A1* expression level in Log_2_ (TPM + 1). **d–f** The correlation between the relative abundance of protein and mRNA of *SLC31A1* in liver cancer, lung squamous cell carcinoma, and ovarian cancer, respectively. **g, h** Comparison of SLC31A1 protein expression in tumor tissues and adjacent normal tissues from patients with liver cancer and stomach cancer. *P < 0.05; **P < 0.01; ***P < 0.001; ****, *P* < 0.0001

Based on datasets from the HPA, GTEx, and FANTOM5 (function annotation of the mammalian genome), *SLC31A1* was found to be widely expressed in many tissues, including the liver, gallbladder, the gastrointestinal tract (such as the small intestine and duodenum) (Fig. 1a; Additional file 1: Figures S1a, b, and c). The protein expression profile in Fig. 1b showed that SLC31A1 had a higher expression in the hippocampus, lung, endometrium, and kidney, and a lower expression in the esophagus, prostate, and skin. Additionally, single-cell RNA-seq analysis revealed high expression of *SLC31A1* in prostatic glandular cells, serous glandular cells, and hepatocytes (Additional file 1: Fig. S1d). It was also noted that *SLC31A1* was also highly expressed in macrophages.

We further examined the expression pattern of *SLC31A1* in tumor tissues. In comparison to corresponding normal tissues, the expression of *SLC31A1* mRNA was increased in most tumor tissues (Fig. 1c). Tumor tissues of breast invasive carcinoma (BRCA), esophageal carcinoma (ESCA), pheochromocytoma, paraganglioma (PCPG), glioblastoma multiforme (GBM), stomach adenocarcinoma (STAD), and uterine corpus endometrioid carcinoma (UCEC) had higher *SLC31A1* expression levels when compared to corresponding normal tissues (all *P* < 0.01). In contrast, decreased *SLC31A1* mRNA expression levels were observed in cholangiocarcinoma (CHOL), kidney chromophobe (KIRC), kidney renal clear cell carcinoma (KIRP), liver hepatocellular carcinoma (LIHC), lung adenocarcinoma (LUAD), lung squamous cell carcinoma (LUSC), prostate adenocarcinoma (PRAD), thyroid carcinoma (THCA) tumor tissues *(P* < 0.001). No significant change in *SLC31A1* expression was found in some tumor types such as pancreatic adenocarcinoma (PAAD) and uterine carcinosarcoma (UCS).

We then evaluated the possible impact of altered mRNA expression on the SLC31A1 protein. Figure 1d–f with relative protein abundance data from cProSite showed a moderate positive correlation between expression levels of SLC31A1 protein and mRNA in liver cancer, lung squamous cell carcinoma, and ovarian cancer. In addition, the relative abundance of SLC31A1 protein in liver cancer and stomach cancer showed a significant difference between tumor tissues and adjacent normal tissues (*P* < 0.0001) (Fig. 1g, h), in line with the mRNA expression difference. These results further confirmed that abnormal SLC31A1 expression might be involved in multiple cancers.

### Association of *SLC31A1* expression with cancer prognosis

**Fig. 2:**
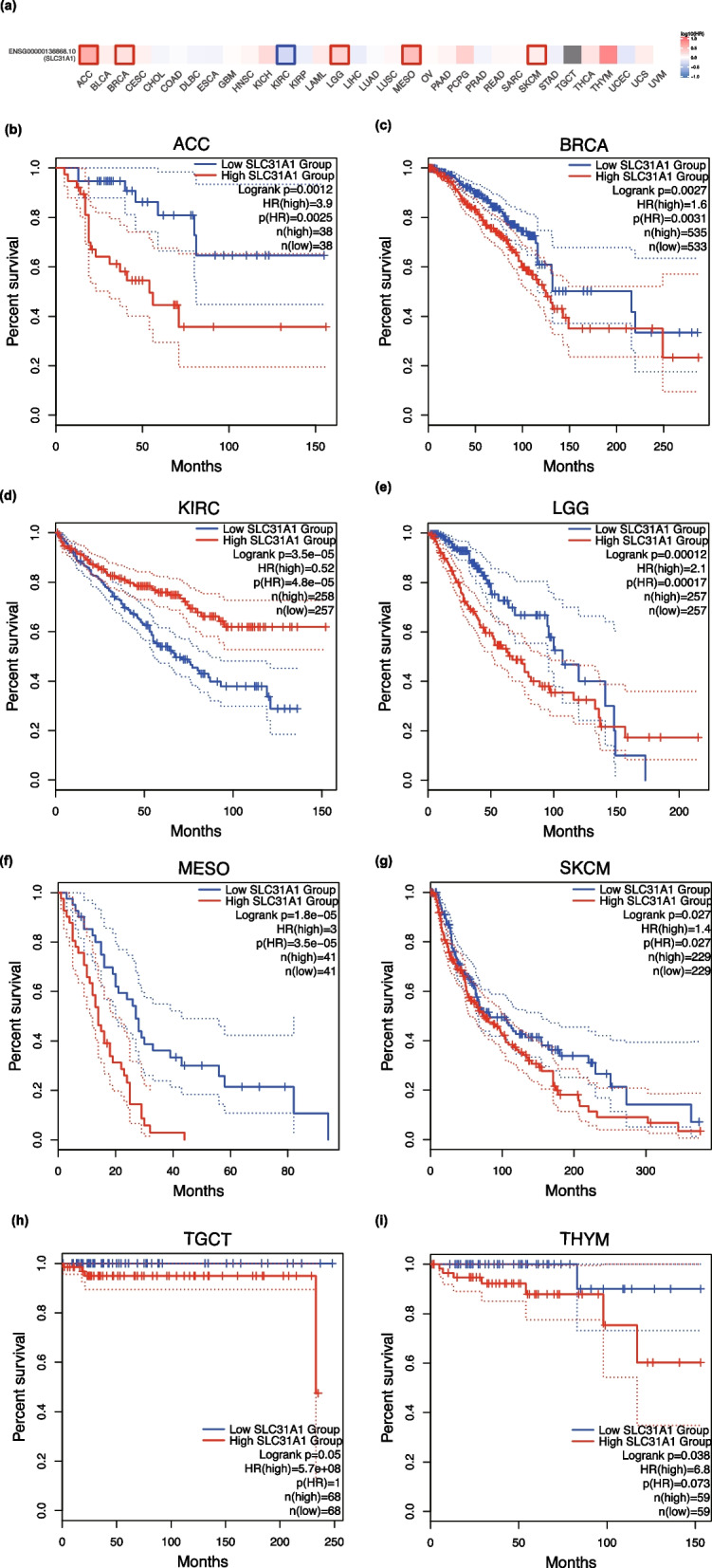
Effects of *SLC31A1* expression on overall survival in different TCGA tumor types. **a** An overview survival map and overall survival analyses **b–i** are derived from GEPIA2. Kaplan–Meier plots of cancer types with significant *p*-values are shown. The 95% confidence intervals of overall survival are indicated by red and blue dotted lines for high and low *SLC31A1* expression groups, respectively. The color of the squares indicates the value of hazzard ratio (HR). Squares with bold outlines in the survival map denote p(HR) < 0.05

**Fig. 3:**
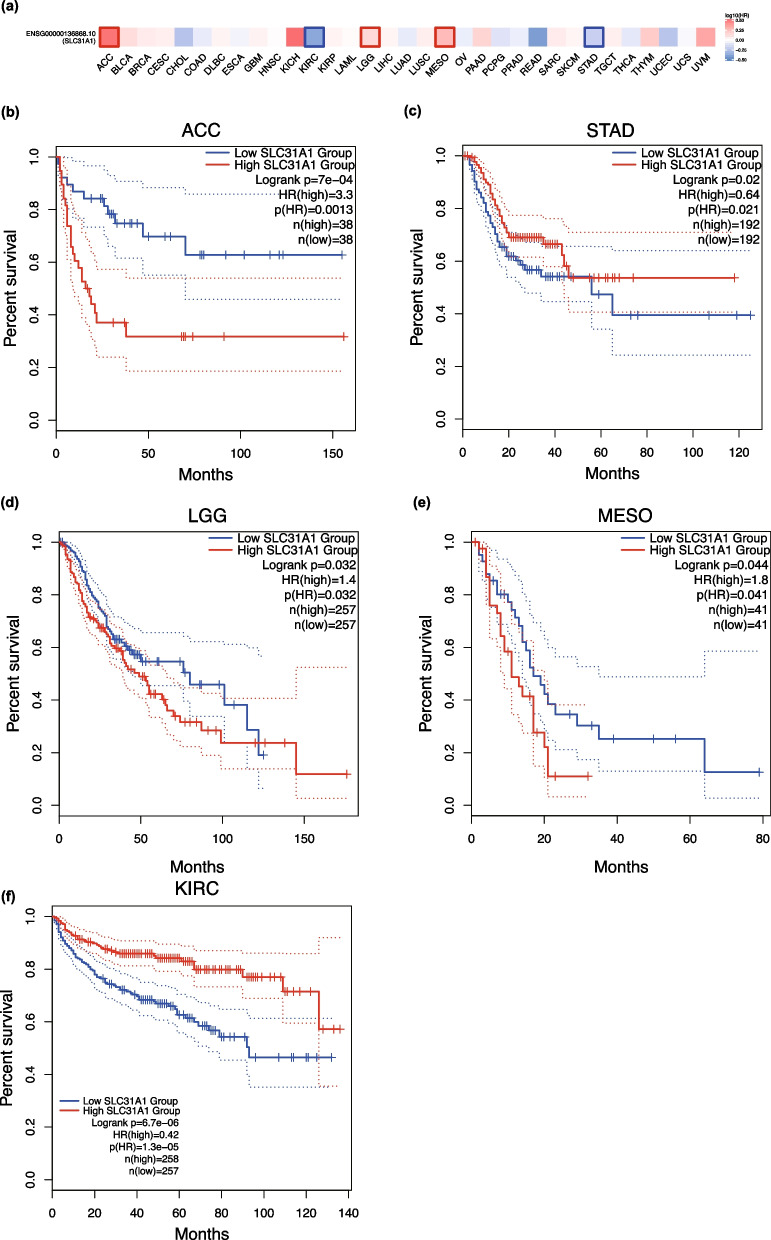
Effects of *SLC31A1* expression on disease-free survival in different TCGA tumor types. GEPIA2 is used to build a survival map (**a**) and conduct disease-free survival **b–f** analyses. Kaplan–Meier plots of cancer types with significant results are displayed. The 95% confidence intervals of disease-free survival are indicated by red and blue lines for the high and low *SLC31A1* groups, respectively. The color of the squares indicates the value of the hazzard ratio (HR). Squares with bold outlines in the survival map denote p(HR) < 0.05

Based on TCGA datasets, GEPIA2 was used to investigate the correlation between *SLC31A1* expression and prognosis in different tumor types. Worse OS was found to be associated with higher *SLC31A1* expression in adrenocortical carcinoma (ACC) (*P* = 0.0012), BRCA (*P* = 0.0027), mesothelioma (MESO) (*P* = 1.8 × 10^–5^), Skin cutaneous melanoma (SKCM) (*P* = 0.027), LGG (*P* = 0.00012), Testicular germ cell tumors (TGCT) (*P* = 0.05), and Thymoma (THYM) (*P* = 0.038), and associated with lower *SLC31A1* expression in KIRC (*P* = 3.5 × 10^–5^) in 5 years (Fig. 2). Additionally, DFS analysis showed that patients with ACC (*P* = 7 × 10^–4^), LGG (*P* = 0.032), and MESO (*P* = 0.044) had worse outcomes if their SLC31A1 levels were higher, while patients with KIRC (*P* = 6.7 × 10^–6^) and STAD (*P* = 0.02) in 5 years had lower levels (Fig. 3). According to the results, abnormal *SLC31A1* expression was associated with poor prognosis in several tumor types.

Furthermore, we used GEPIA2 to examine the association between *SLC31A1* expression and pathological stages of tumors and found a significant difference of *SLC31A1* expression among pathological stages of ACC, KIRC, and MESO (all *P* < 0.05) (Additional file 2: Figure S2).

### *SLC31A1* genetic alterations in tumors

**Fig. 4:**
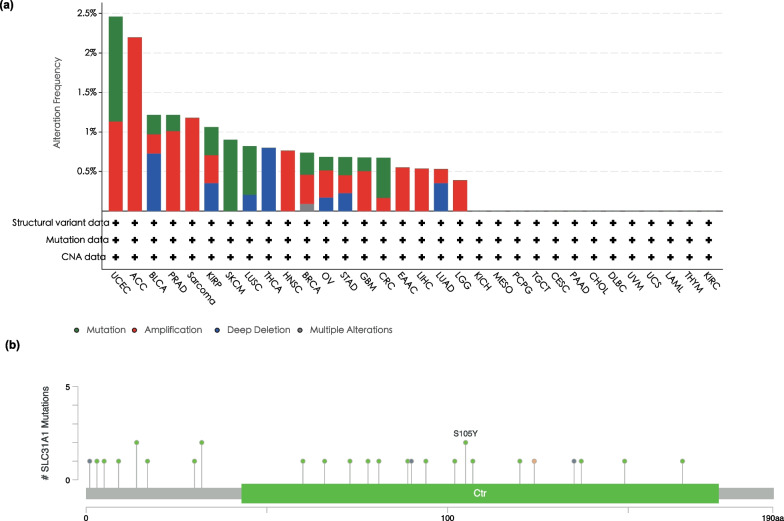
*SLC31A1* genetic alteration in various tumor types in TCGA. The frequency (**a**) and position of *SLC31A1* mutation site **b** are derived from cBioPortal

CBioPortal was then utilized to examine *SLC31A1* gene alterations in TCGA datasets of various tumor types. It was found that tumor samples from UCEC had the highest *SLC31A1* genetic alternation frequency (2.46%). In ACC tumor samples, all *SLC31A1* mutations were copy number amplified (Fig. 4a; Additional file 4: Table S1), which in all tumor samples from TCGA were the most common genetic alterations  in *SLC31A1*. Besides UCEC and ACC, genetic alteration of *SLC31A1* was observed in more than 1% of Bladder Urothelial Carcinoma (BLCA), Prostate, Adenocarcinoma, Sarcoma, and Kidney Renal Papillary Cell Carcinoma*.* As shown in Fig. 4b, a total of 27 *SLC31A1* mutations, including 23 missense mutations, one fusion mutation, two frame-shift mutations, and one translation start-codon mutation, were contained in TCGA tumor samples (Additional file 4: Table S2). According to the TCGA tumor samples, S105Y is the most prevalent point mutation in *SLC31A1* (Fig. 4b). In the ICGC database, the mutation S105Y was also the most prevalent mutation  in *SLC31A1* (Additional file 3: Figure S3).

Furthermore, by using the database of OncoVar and COSMIC, S105Y was identified as a potential driver mutation with an OncoVar-score of 0.998 using the AI-driver method (Additional file 4: Tables S3, S4).

### Correlation between *SLC31A1* expression and immune infiltration

**Fig. 5:**
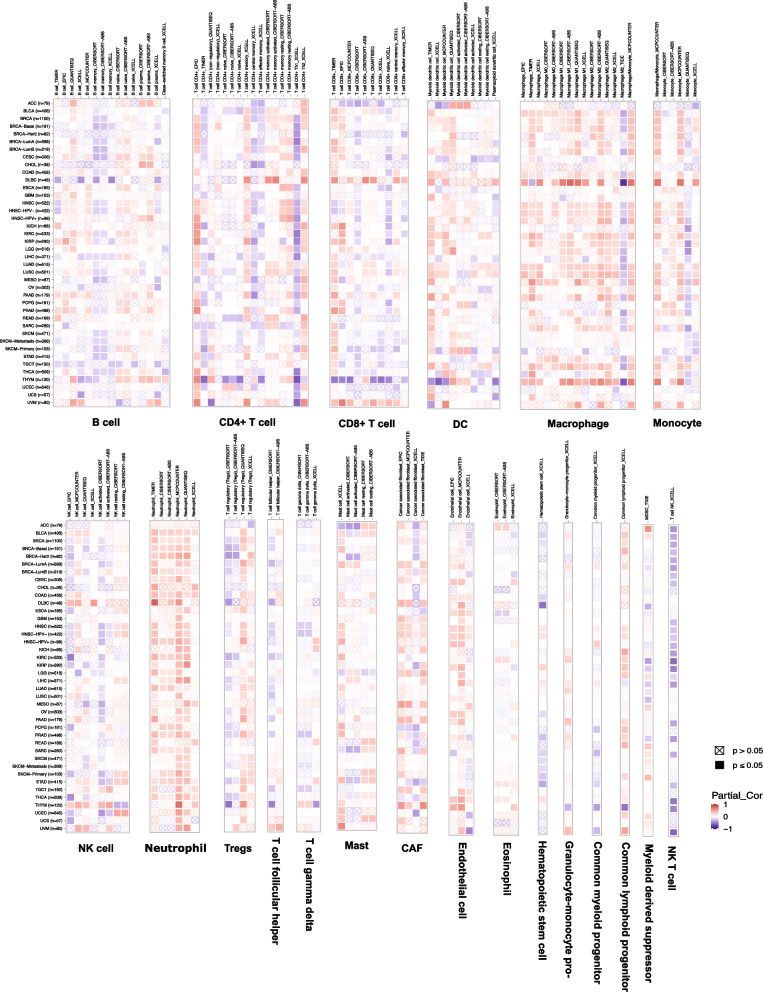
The correlation of *SLC31A1* and the infiltration levels of B cells, cancer associate fibroblast, common lymphoid progenitor, common myeloid progenitor, DC, endothelial cells, eosinophil, granulocyte-monocyte progenitor, hematopoietic stem cells, macrophage, mast cells, monocyte, myeloid-derived suppressor cells, neutrophil, NK cells, CD4 + T cells, CD8 + T cells, T cell follicular helper, T cell gamma delta, NK T cells, and Tregs. Positive correlation in red and negative correlation in blue.

The immune infiltration of tumors could influence the prognosis and treatment. We used TIMER2 to explore the correlation of *SLC31A1* in 21 immune infiltrates in multiple tumors using algorithms including TIMER, xCell, MCP-counter, CIBERSORT, EPIC, and quanTIseq (Fig. 5). Notably, *SLC31A1* was positively correlated with macrophage infiltration in multiple tumors including BLCA, COAD, HNSC, KIRC, LUAD, LUSC, PAAD, and THYM. *SLC31A1* was positively correlated with neutrophil infiltration in BLCA, COAD, and STAD. Such findings indicated a potential role of *SLC31A1* in the immune process during cancer development and progression.

### Enrichment of *SLC31A1*-related genes in metabolic pathways

**Fig. 6:**
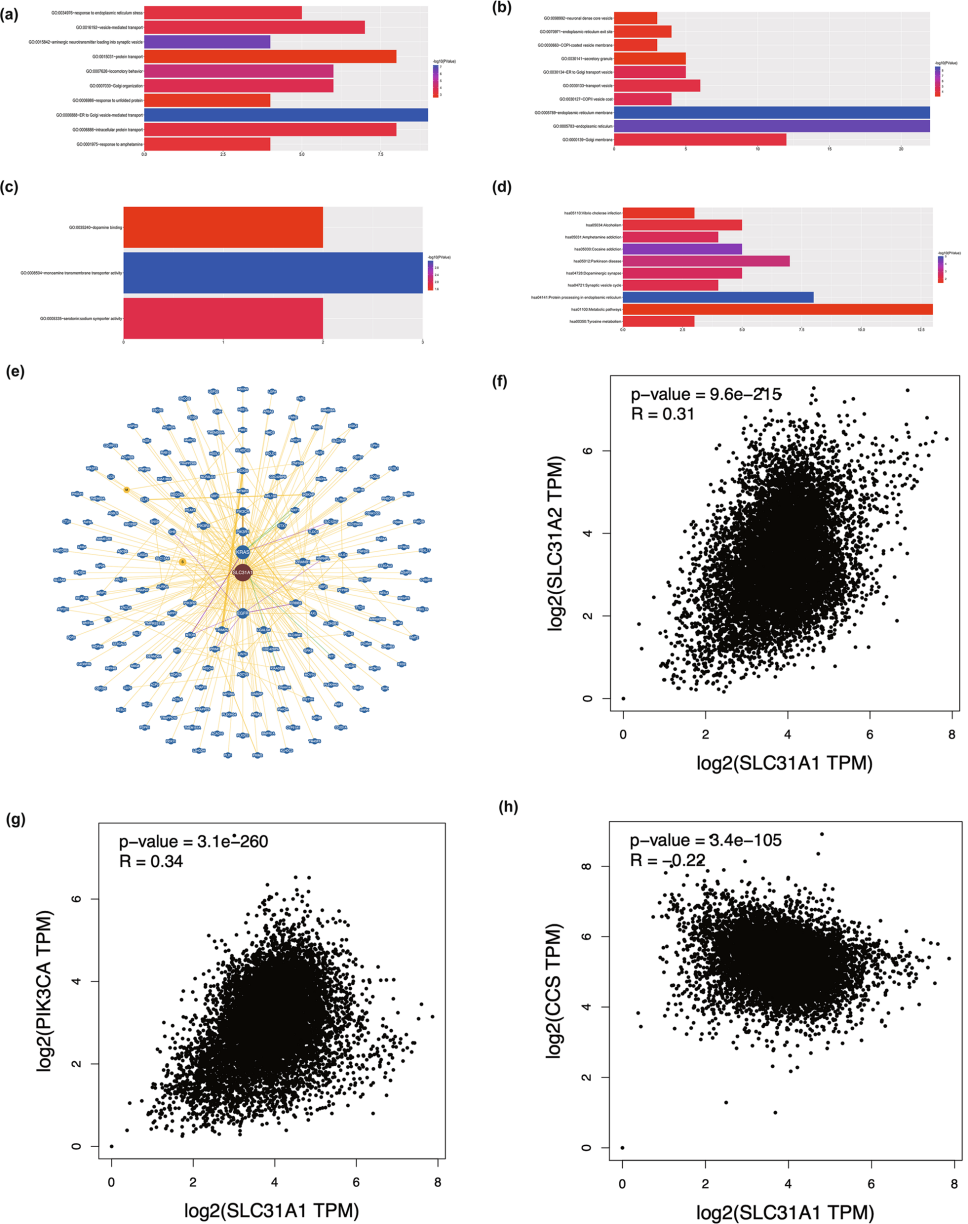
*SLC31A1*-related gene enrichment analysis.** a–d** Gene Ontology (GO) analysis and Kyoto Encyclopedia of Genes and Genomes (KEGG) analysis of the top 100 genes co-expressed with *SLC31A1* ranked by Pearson correlation coefficient from GEPIA2. **e** C1ORF112-protein interactions obtained from BioGRID. **f–h** Correlation analysis between *SLC31A1* and *CCS* and Erb-B2 Receptor Tyrosine Kinase 3 (*ERBB3*) is conducted by GEPIA2 across all tumor samples from TCGA

GEPIA2 was used to extract the top 100 genes with expression patterns similar to *SLC31A1* in all tumor types from TCGA to investigate the gene’s functional impact. (Additional file 4: Table S5). GO and KEGG enrichment analysis indicated that these genes were involved in metabolic pathways and protein processing in the endoplasmic reticulum (Figs. 6a–d). These findings prompted us to wonder whether *SLC31A1* plays a role in these biological processes by interacting with essential proteins involved in protein binding, integral components of the membrane, metabolic pathways, and protein processing in the endoplasmic reticulum (Additional file 4: Tables S6-9). Figure 6e showed the PPI network which was conducted in BioGRID (minimum evidence = 1). Based on the Wikipathways annotation, three genes in the copper homeostasis pathway including copper chaperone for superoxide dismutase (*CCS*), Phosphatidylinositol-4,5-bisphosphate 3-kinase Catalytic subunit alpha (*PIK3CA*) and Solute Carrier Family 31 Member 2 (*SLC31A2*) were found as nodes in the PPI network [32]. Furthermore, the expression level of *SLC31A2*, *PIK3CA,* and *CCS* was correlated with *SCL31A1* (Fig. 6f, g) (Spearman *r* = 0.31, 0.34, and −0.22, respectively).

## Discussion

The multi-omics data of 33 tumor types from the TCGA project allow pan-cancer analyses of biomarkers and therapeutic targets using bioinformatic and statistic tools [7, 33–36]. Our current study evaluated the clinical significance of *SCL32A1*, a key cuproptosis-regulatory gene, in various cancer types and implicated a potentially substantial role of cuproptosis in cancers.

The recently reported pathophysiological role of cuproptosis may provide new insight into anticancer treatments. *SLC31A1* is the primary regulator of Cu uptake, and it expresses in most cells [37, 38]. Cuproptosis is a novel mechanism of cell death whose core is the tricarboxylic acid cycle, and it relies on the mitochondrial respiration [14]. *SLC31A1* has recently been proposed as a biomarker for cancer therapy and could play a role in chemoresistance in a few types of cancers [39, 40]. High-affinity copper uptake protein 1 (CTR1) encoded by *SLC31A1* is the primary component responsible for Cu uptake in cells [41, 42]. A recent study revealed that CTR1 could function as a redox sensor to drive neovascularization [43]. A strong correlation between CTR1 and Programmed death-ligand 1 paved the way for clinical trials to evaluate Cu chelators as immune checkpoint inhibitors [44]. The current study comprehensively explored whether *SLC31A1* plays a role in multiple tumors.

In the current study, TCGA datasets showed that *SLC31A1* was expressed in various tissues. According to our findings, dysregulation of the *SCL31A1* gene was associated with clinical parameters or prognosis in multiple types of cancer. It was found that a high expression level of *SLC31A1* in ACC, KIRC, LGG, and MESO was associated with poor OS and DFS. More and more evidence has shown that genomic mutations influence tumor progression and chemotherapy response [45–47]. For example, there is evidence that genetic polymorphisms of *SLC31A1* are associated with chemotherapy resistance and clinical outcomes in cancer patients [48]. In the current study, UCEC (> 2%) had the highest mutation rate of *SLC31A1*, followed by ACC, BLCA, and PRAD. Based on these, *SLC31A1* has been found to act as an oncogene in the progression of numerous cancers and may serve as a useful predictor of cancer prognosis.

The molecular mechanism of *SLC31A1* in cancers remains to be elucidated. Our results indicated that *SLC31A1* might contribute to changes in the immune microenvironment in cancer tissues. The immune microenvironment has also been found to influence molecular phenotypes and prognoses [49–52]. Our results showed positive correlations of *SLC31A1* expression with neutrophil and macrophage infiltration in several tumor types. Such correlation with macrophage infiltration was in line with the high expression level of *SLC31A1* in macrophages in single-cell RNA-Seq data, emphasizing the importance of *SLC31A1* and the related cuproptosis in the cancer-related immune process. And neutrophils were reported to be involved in the metastasis of breast cancer [53].

Our gene enrichment analysis showed that there was a strong correlation between genes that co-express with *SLC31A1* and metabolic pathways in the endoplasmic reticulum. In particular, *SLC31A2* and *PIK3CA* were copper homeostasis-regulated genes with a key role in tumor. Recently the function of *SLC31A2* has been reported to associate with the development of lung adenocarcinoma, ovarian carcinoma, hepatocellular carcinoma, and sensitivity to Cisplatin [54–57]. In the aspect of copper regulation, *PIK3CA* was reported to be relative to glioma, breast cancer, and medulloblastoma [58–60]. Our results suggested that *SLC31A1* may play a key role in cancer by influencing metabolic and Cu-related processes.

Our preliminary findings suggest that *SLC31A1* could be involved in a variety of tumor types. Nevertheless, there are limitations in the current study. For some rare tumor types, the sample sizes were relatively small and our finding needed to be validated in independent cohorts. Further studies are warranted to determine the molecular function of *SLC31A1* in tumorigenesis.

## Conclusions

Our pan-cancer analysis demonstrates that the cuproptosis-regulatory gene *SLC31A1* is dysregulated in various cancers with its expression and genetic alteration associated with clinical outcomes in patients with these tumors. Additionally, immune infiltration analysis and gene enrichment analysis provide new insight into potential mechanisms related to *SLC31A1* in cancers. Our study thus warrants further experimental and clinical studies to understand the function of *SLC31A1* and its potential practical applications in cancer therapy and prognosis prediction.

## Supplementary Information

**Additional file 1**.** Fig. S1**: *SLC31A1 *expression status in different normal tissues.** a**,** b**, and** c** tissue expression profiles of *SLC31A1* based on datasets of the GTEx, FANTOM5 (Function annotation of the mammalian genome 5), and HPA dataset.** d*** SLC31A1* expression in various cell types.

**Additional file 2**.** Fig. S2**. Correlation between *SLC31A1* expression and pathological stages of ACC, KIRC, and MESO from TCGA datasets. *SLC31A1* expression is in Log2 (TPM + 1).

**Additional file 3**.** Fig. S3**.** a** The mutation S105Y in the database of ICGC.** b** The top 10 cancer distribution of donors with S105Y from different cohorts. Donors affected: donors in the current project with *SLC31A1* affected by simple somatic mutation (SSM)/SSM-tested donors in the current project. LMS-FR: Soft Tissue cancer (France), BTCA-SG: Biliary Tract cancer (Singapore), SKCA-BR: Biliary Tract cancer (Brazil), MELA-AU: Skin cancer (Australia), LIRI-JP: Liver cancer (Japan), ESAD-UK: Esophageal cancer (United Kingdom), UTCA-FR: Uterine cancer (France), NACA-CN: Nasopharyngeal cancer (China), LICA-CN: Liver cancer (China).

**Additional file 4**.**Table S1**: *SLC31A1* genetic alteration types in TCGA datasets from cBioportal.** Table S2**. *SLC31A1* genetic mutations summary in TCGA datasets from cBioportal.** Table S3**. *SLC31A1* genetic alteration profile.** Table S4**: The source of the mutation of *SLC31A1*.** Table S5**: Top 100 genes with similar expression patterns to the *SLC3A1* gene from all tumor types of TCGA datasets by GEPIA2.** Table S6**: GO cellular component (CC) enrichment analysis of 100 *SLC31A1*-correlated genes.** Table S7**: GO biological process (BP) enrichment analysis of 100 *SLC31A1*-correlated genes.** Table S8**: GO molecular function (MF) enrichment analysis of 100 *SLC31A1*-correlated genes.** Table S9**: KEGG enrichment analysis of 100 *SLC31A1*-correlated genes.

## Data Availability

The data used to support the findings of this study are available from the corresponding author upon request.

## Abbreviations

SLC31A1: Solute carrier family 31 member 1
PPI: Protein–protein interaction
ACC: Adrenocortical carcinoma
BLCA: Bladder urothelial carcinoma
BRCA: Breast carcinoma
CHOL: Cholangiocarcinoma
ESCA: Esophageal carcinoma
LGG: Low-grade glioma
MESO: Mesothelioma
PCPG: Pheochromocytoma and paraganglioma
GBM: Glioblastoma multiforme
STAD: Stomach adenocarcinoma
SKCM: Skin cutaneous melanoma
TGCT: Testicular germ cell tumors
THYM: Thymoma
UCEC: Uterine corpus endometrioid carcinoma
KIRC: Kidney chromophobe
KIRP: Kidney renal clear cell carcinoma
LIHC: Liver hepatocellular carcinoma
LUAD: Lung adenocarcinoma
LUSC: Lung squamous cell carcinoma
PRAD: Prostate adenocarcinoma
THCA: Thyroid carcinoma
PAAD: Pancreatic adenocarcinoma
UCS: Uterine Carcinosarcoma
OS: Overall survival
DFS: Disease-free survival
GO: Gene ontology
KEGG: Kyoto Encyclopedia of Genes and Genomes
ICGC: International Cancer Genome Consortium
TCGA: The Cancer Genome Atlas
FANTOM5: Function annotation of the mammalian genome 5
CCS: Copper chaperone for superoxide dismutase
SLC31A2: Solute carrier family 31 member 2
PIK3CA: Phosphatidylinositol-4,5-bisphosphate 3-kinase catalytic subunit alpha
