# Supplementary figures and images for: Systematic pan-cancer analysis identifies SLC31A1 as a biomarker in multiple tumor types
Source: BMC Med Genomics. 2023 Mar 27;16:61. doi: 10.1186/s12920-023-01489-9 (PMC10041742; doi:10.1186/s12920-023-01489-9)

(a)

GTEx dataset

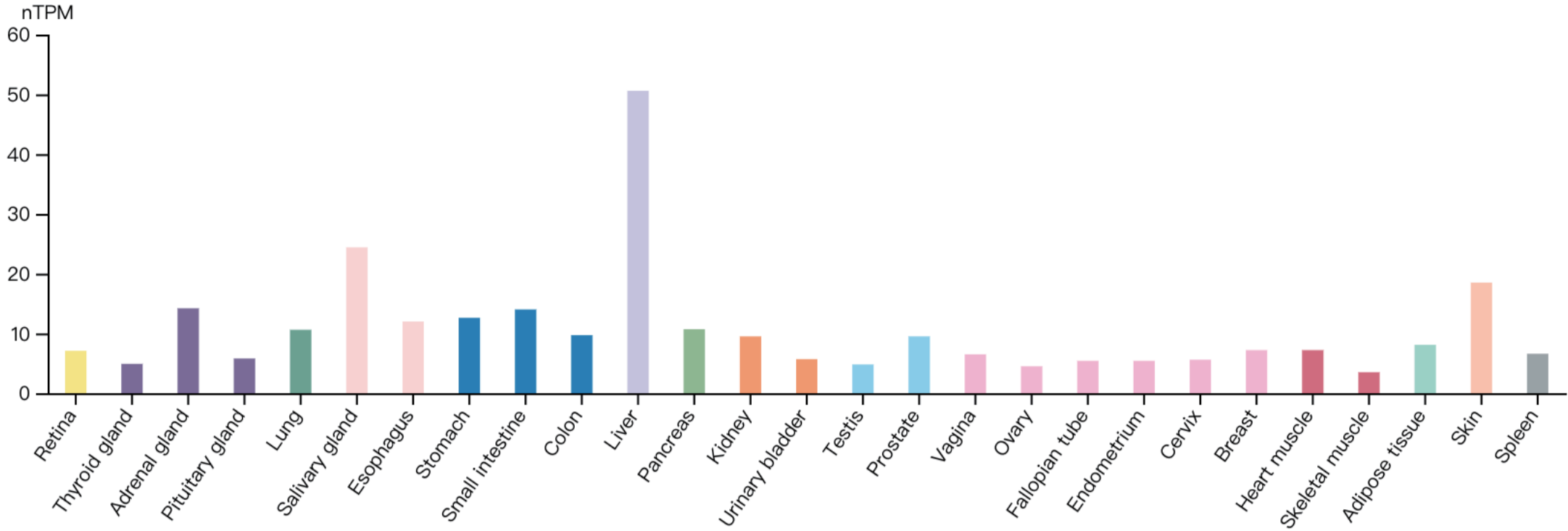

(b)

FANTOM5 dataset

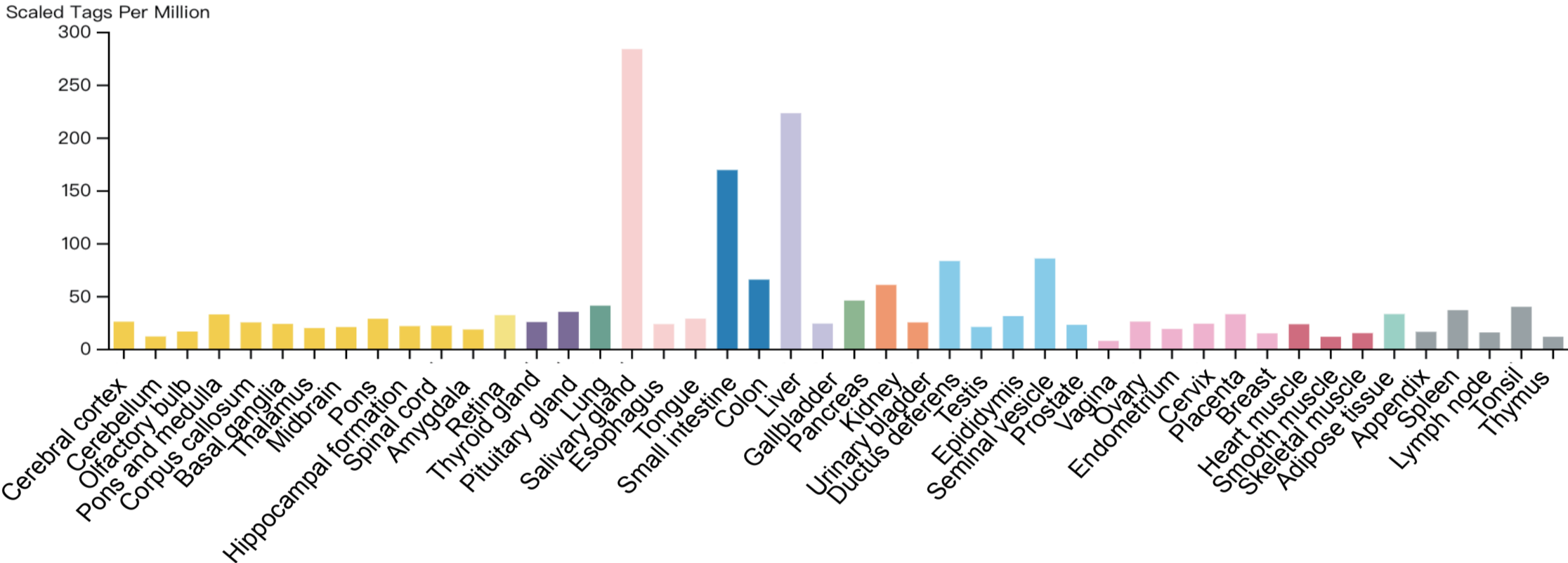

(c)

HPA dataset

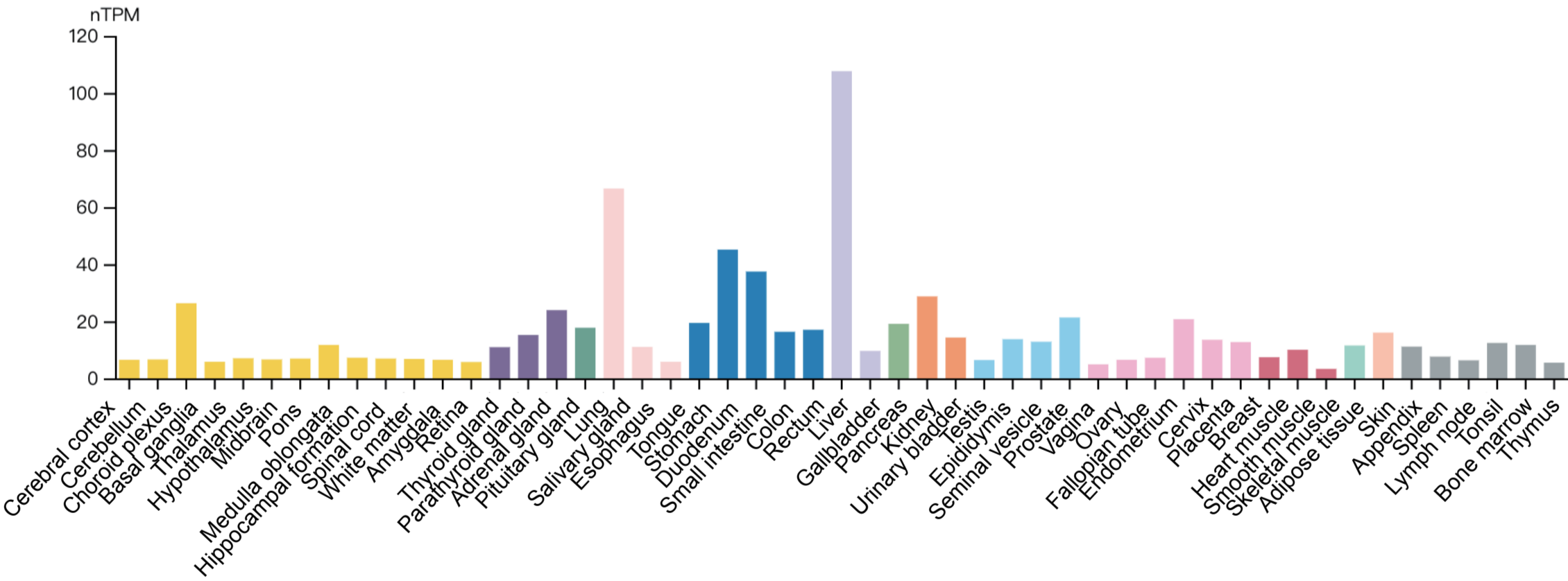

(d)

Single cell types

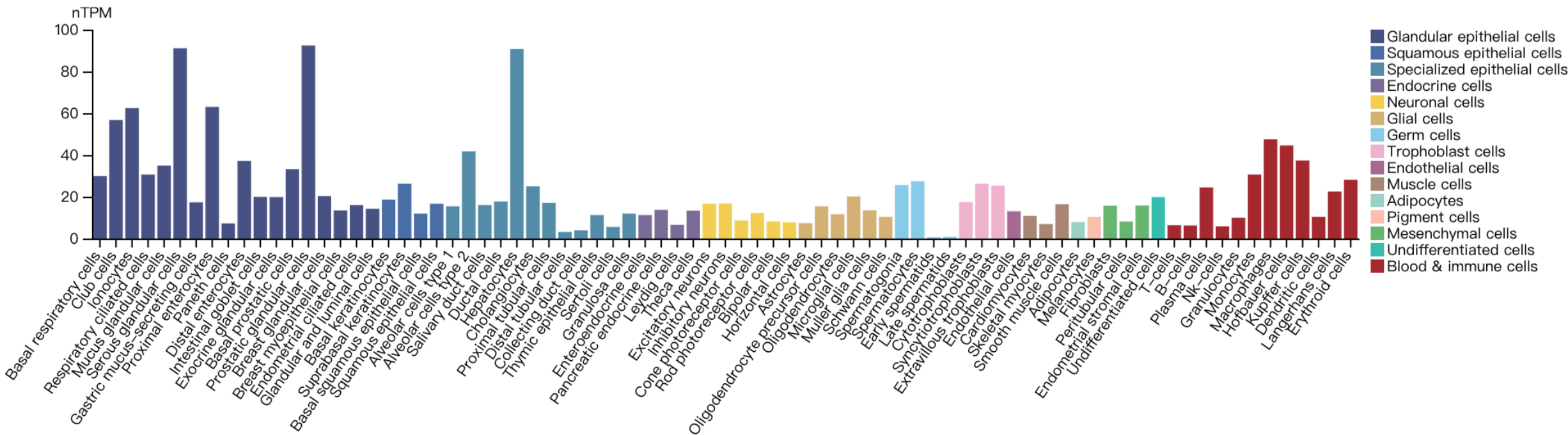

Supplement: Supplementary file 1 — Additional file 1. Fig. S1: SLC31A1 expression status in different normal tissues. a, b, and c tissue expression profiles of SLC31A1 based on datasets of the GTEx, FANTOM5 (Function annotation of the mammalian genome 5), and HPA dataset. d SLC31A1 expression in various cell types. [file 12920_2023_1489_MOESM1_ESM.pdf]

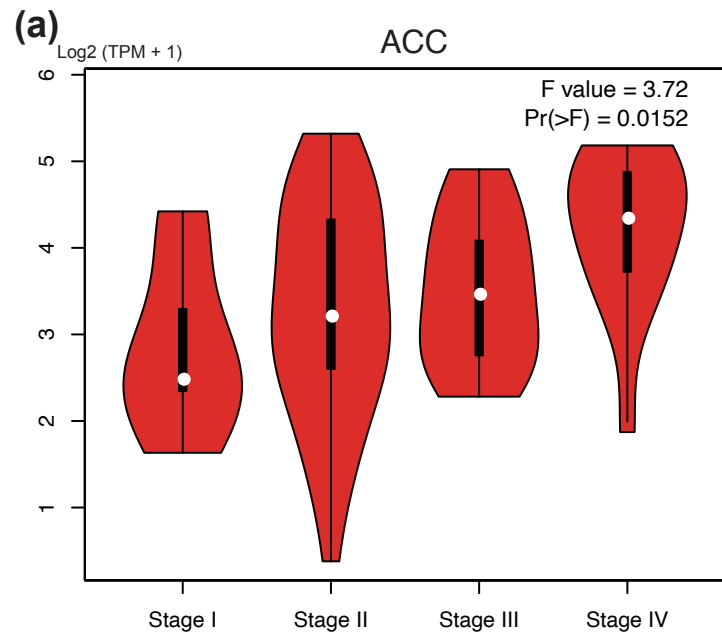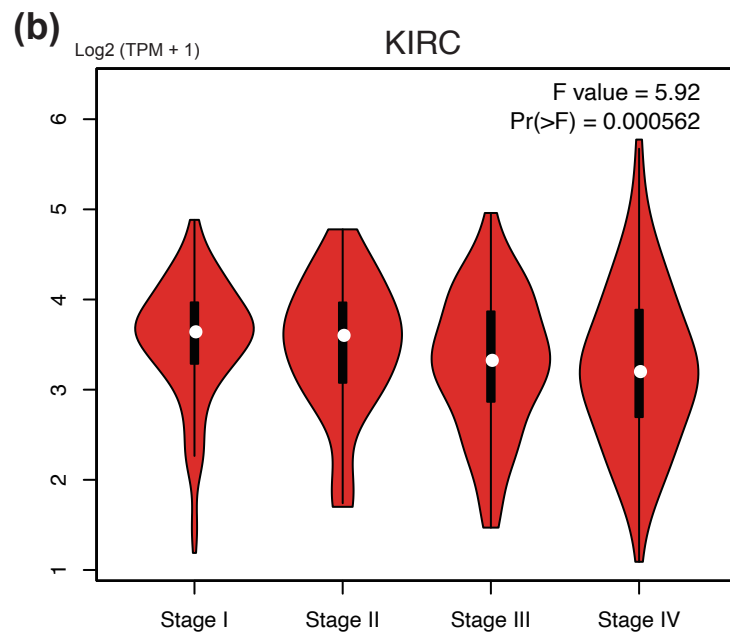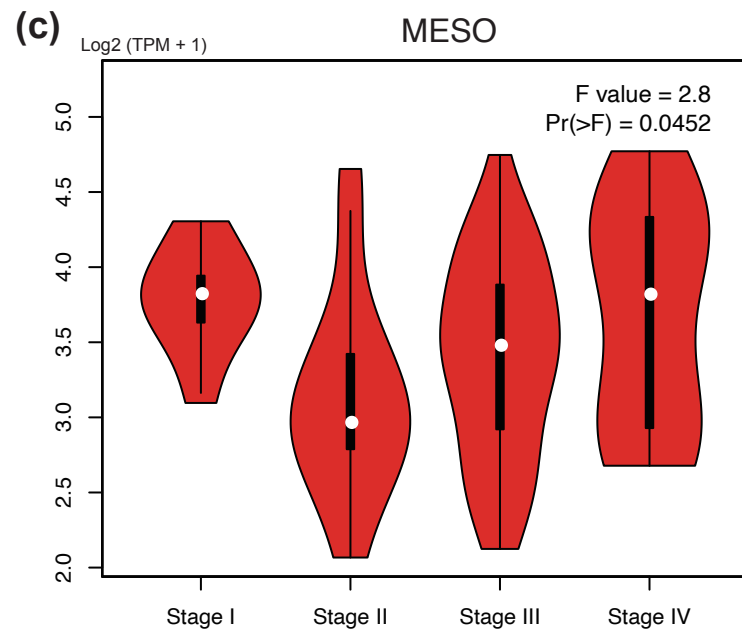

Supplement: Supplementary file 2 — Additional file 2. Fig. S2. Correlation between SLC31A1 expression and pathological stages of ACC, KIRC, and MESO from TCGA datasets. SLC31A1 expression is in Log2 (TPM + 1). [file 12920_2023_1489_MOESM2_ESM.pdf]

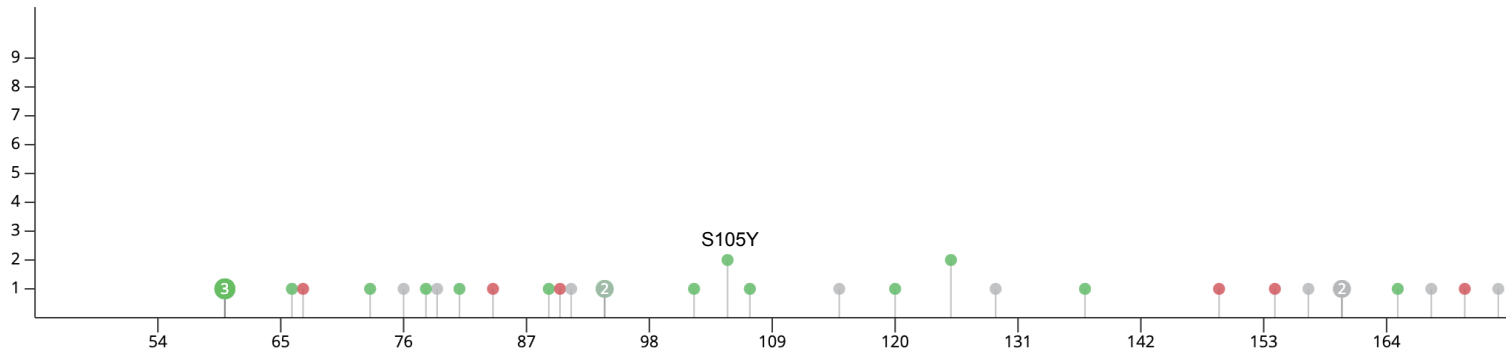

Supplement: Supplementary file 3 — Additional file 3. Fig. S3. a The mutation S105Y in the database of ICGC. b The top 10 cancer distribution of donors with S105Y from different cohorts. Donors affected: donors in the current project with SLC31A1 affected by simple somatic mutation (SSM)/SSM-tested donors in the current project. LMS-FR: Soft Tissue cancer (France), BTCA-SG: Biliary Tract cancer (Singapore), SKCA-BR: Biliary Tract cancer (Brazil), MELA-AU: Skin cancer (Australia), LIRI-JP: Liver cancer (Japan), ESAD-UK: Esophageal cancer (United Kingdom), UTCA-FR: Uterine cancer (France), NACA-CN: Nasopharyngeal cancer (China), LICA-CN: Liver cancer (China). [file 12920_2023_1489_MOESM3_ESM.pdf]
